# Cure of Cleft Palate

**Published:** 1890-12

**Authors:** Henry O. Marcy

**Affiliations:** Boston, Mass.


### THE DENTAL REGISTER.

VOL. XLIV.] DECEMBER, 1890. [No. 12.

### COMMUNICATIONS.

### Cure of Cleft Palate by a Double Flap Operation and Closure with the Buried Tendon Suture.

BY HENRY O. MARCY, M.D., BOSTON, MASS.

Read in the Section of Dental and Oral Surgery at the forty-first annual meeting of the American Medical Association, at Nashville, Tenn., May, 1890.

Nearly all surgical operations have had their distinctive modifications, with surprising gain in the result, based upon the knowledge of the role of bacteria in wounds. In large measure, however, the surgery of the mouth, the throat, and the nasal passages, has proved an exception, since all wounds of these tissues, as ordinarily treated, must be looked upon as extremely liable to become infected from the ever-present bacteria, existing normally in the mucous secretions of the tract, and necessarily conveyed to it in respiration and in the taking of food.

Dentistry, as an art, may be said to have commenced with the present generation ; as a science, within the last few years, since by the labors of Miller, Nelson, Andrews, and others, the demonstration is ample that dental decay is dependent upon bacterial development within the structures of the teeth. All scientific observers now agree that the preservation of a diseased tooth is dependent upon the removal of the infected portion, and the treatment of the member in such a way as to preserve permanently its tissues aseptic. These principles, when applied to the treatment of a single tooth, are comparatively simple and easy to carry into effect. When the cancellated structure of the jaw, or any of the bones forming the nasal or oral tract are involved, the conditions are much more serious, and the limitations, or removal of the infected parts often form problems very difficult of solution.

Wounds of the soft parts, even when made through comparatively healthy tissues and closed with the greatest nicety by the surgeon at the time of operation, often heal very unsatisfactorily, becoming the seat, at least, of a local infection, and repair either fails entirely, or goes on slowly by the processes of granulation. This has its explanation from the great difficulty of excluding infective material from the wounded surfaces, and the reason why general septic infection with its attendant danger is not more common is found in the extraordinary vascularity affording a better protective nutrition of the parts involved.

Plastic surgery of the oral cavity has made comparatively little advance, for the above reasons, although it is almost entirely revolutionized in other parts of the body by modern surgical procedure.

Operative measures for the relief and cure of cleft palate generally fail on account of non-closure of the wound, dependent upon infection and difficulty of the retention of the parts at rest, especially at its free, or distal extremity. During the last few years the attempt at repair is less often made by the surgeon than formerly, doubtless, in large measure due to the extremely ingenious and useful mechanical devices in the way of artificial plates, one of the distinctive triumphs of American mechanical dentistry.

I confess myself to have given over the field as one entirely unfruitful of surgical relief until within the last year, when I carried into effect, in a single case, the method which forms the subject of this brief contribution.

Miss J; age 25; a strong and healthy woman, has a congenital cleft of the soft palate with only slight indentation of the bony structures. Each half of the uvula is well developed and by moderate tension the edges of the cleft can be brought into apposition. After a careful explanation of the intended procedure the patient gladly availed herself of the hoped-for results from operation. Aided by Dr. Charles Bullock and Dr. S. N.

Nelson, I commenced the operation by the introduction of a tracheotomy tube. With careful washing and irrigation the nasal and oral passages were rendered aseptic, using freely a sublimate solution of strength 1–1000. After the introduction of the mouth-gag and packing the pharynx with an aseptic sponge, the soft palate was seized upon either side, the edges slightly refreshed with scissors and split laterally to the depth of about one-third of an inch. The posterior flaps thus formed were carefully joined by a very fine over-and-over tendon suture. A somewhat larger suture, commencing at the angle at the base of the division of the flap, was carried laterally from side to side through the centre of the soft palate, a little beyond the line of lateral division. This line of sutures extended quite to the uvula. The approximation of the sides of the wound necessarily everted the edges and by so much increased the fresh surfaces brought in contact, while at the same time it completely buried the line of sutures. The everted edges presenting in the roof of the mouth were then carefully joined by a very fine continuous tendon suture. Two double-looped stitches, taken as far away from the central line as possible, were then introduced in order to prevent any lateral strain upon the central wound. The mouth and nose were closed, and only rarely required to be cleansed of mucus during the four subsequent days; after cleansing they were dusted with iodoform by means of the iodoform blower. Respiration went on comfortably through the tracheal tube. A large injection of weak beef tea, given once in six hours, served the purpose of food and drink. The wound remained non-infected, and repair ensued as in aseptic wounds in other parts of the body, with the exception of the tip of the uvula, where a small sluff formed from over-constriction of the suture.

The above case is reported as an experimental study, and is at least instructive in giving possibilities of better surgical results than those generally obtained in this most distressing deformity. If such seeming heroic procedure as antecedent tracheotomy may meet the approval of the profession is a question for discussion. Without it all attempt at rigid antiseptic conditions of the parts is seemingly futile. External wounds of the body are readily sealed from bacterial infection by the simple application of iodoform collodion, but thus far all attempts have failed in protecting mucous surfaces by germ-proof dressings, and we are under the necessity of making the cavity itself aseptic, and maintaining it in this condition until repair can ensue, or of accepting the dangers of infection which, when respiration is allowed to go on in the usual manner, generally gives failure in result.

The advantages of the use of the tendon suture in this operation appear to be equal to those claimed for it in operative wounds in other parts of the body, and if this is true, it is the only suture material to be recommended for this operation. If it shall happen to me to attempt again the repair of these parts a modification of instruments would simplify in a considerable degree the operation. Forceps bent at a suitable angle, the blades protected by rubber tubing to prevent injury of the enclosed parts, would greatly facilitate the seizure and holding of the flap preparatory to splitting it. The division also can be effected easier by a knife, the blade of which is at a suitable angle to the handle. The needle forceps also need special adaptation of the curve for the easy introduction of the sutures. Any practical surgeon, however, can effectually operate with the instruments ordinarily at his disposal.

DISCUSSION.

Dr. John S. Marshall, of Chicago, said: At the request of the secretary of the section I have consented to open the discussion upon this paper, and through the courtesy of Dr. Marcy I have had an opportunity to give it a somewhat careful reading. I consider the paper a valuable one in that it presents an entirely new method of operation for closing cleft palate. Whether it will revolutionize the common practice in this operation time and experience only can establish. It is, however, a step in the right direction, as it looks toward establishing and maintaining an aseptic condition of the mouth during the operation and the process of repair in the wound.

Those of us who have had experience in operations upon the soft tissues and bones in and around the oral cavity, will appreciate how great a boon thorough aseptic conditions would be in all wounds opening into the mouth, and how difficult it has been to prevent suppuration in compound fractures and other extensive injuries to the maxillary bones. Miller, in his researches, has found more than a hundred forms of micro-organisms in the human mouth, including nearly all the pathogenic forms known to science, while the most cleanly mouths are found to be the constant habitat of a variety of forms. It would therefore seem to be an Herculean task to cleanse the mouth and render it thoroughly aseptic even by the method suggested by the essayist.

It has been my practice for years to combat the tendency to infection by the use of solutions of bichloride of mercury and of carbolic acid of various strengths, the parts being irrigated and sprayed with them at frequent intervals. Dr. Marcy's method, however, could hardly be utalized in cases of severe compound comminuted fractures of the jaw, where many times under the most favorable circumstances it requires from two to three weeks to heel the external wound, and serious results might follow such prolonged tracheal breathing and feeding by the rectum.

In the operation of staphylorraphy I believe the aseptic method will prove of great value, though to the patient tracheotomy and the plugging of the throat and nose will seem very formidable, and will deter a great many from availing themselves of the offered benefits.

With the new features of the operation itself I am still more pleased.

The great difficulties in the way of a successful result in closing of the *vellum palati* have been to obtain perfect coaptation of the freshened edges, the prevention of tension upon the stitches, and perfect rest of the parts. By Dr. Marcy's operation these are largely overcome. In the lateral splitting of the *vellvm* along the border of the cleft the fresh surfaces are greatly increased, and by that much it would seem to add to the chances of successful union.

The method of stitching is unique, and the use of the buried tendon suture adds to the possibility of a successful result, though at first thought it would seem somewhat difficult to accomplish except by instruments specially devised.

The result in the case reported is, however, no better, in fact, not so good as in some cases which have been treated by the old methods of operating, as primary union through the whole extent of the wound is sometimes obtained by them. By the old methods about one in four are failures, but Dr. Marcy's method gives promise of better results, and until such time as it has received a fair trial we should withhold our judgment.

I trust Dr. Marcy will keep the profession advised in regard to all his future efforts in this direction, and I assure you it shall have a fair trial at my hands.

